# An economic way of reducing health, environmental, and other pressures of urban traffic: a decision analysis on trip aggregation

**DOI:** 10.1186/1471-2458-5-123

**Published:** 2005-11-25

**Authors:** Jouni T Tuomisto, Marko Tainio

**Affiliations:** 1 Centre for Environmental Health Risk Analysis, National Public Health Institute (KTL), P.O. Box 95, FI-70701, Finland

## Abstract

**Background:**

Traffic congestion is rapidly becoming the most important obstacle to urban development. In addition, traffic creates major health, environmental, and economical problems. Nonetheless, automobiles are crucial for the functions of the modern society. Most proposals for sustainable traffic solutions face major political opposition, economical consequences, or technical problems.

**Methods:**

We performed a decision analysis in a poorly studied area, trip aggregation, and studied decisions from the perspective of two different stakeholders, the passenger and society. We modelled the impact and potential of *composite traffic*, a hypothetical large-scale demand-responsive public transport system for the Helsinki metropolitan area, where a centralised system would collect the information on all trip demands online, would merge the trips with the same origin and destination into public vehicles with eight or four seats, and then would transmit the trip instructions to the passengers' mobile phones.

**Results:**

We show here that in an urban area with one million inhabitants, trip aggregation could reduce the health, environmental, and other detrimental impacts of car traffic typically by 50–70%, and if implemented could attract about half of the car passengers, and within a broad operational range would require no public subsidies.

**Conclusion:**

Composite traffic provides new degrees of freedom in urban decision-making in identifying novel solutions to the problems of urban traffic.

## Background

Personal car traffic is one of the major sources of particulate matter (PM), a major pollutant that is estimated to be responsible for 300000 premature deaths every year in the European Union [1]. In Finland, the estimates are 920 deaths and 960000 restricted activity days for all PM [1]. The direct PM emissions from bus traffic were estimated to be responsible for 12 deaths per year in the Helsinki metropolitan area in 1999, but bus emissions accounted for less than one fifth of the total road traffic PM emission [2]. Although cars have become cleaner during recent years, the growth of car traffic and the location of the emission near the breathing zone mean that these emissions are still ranked high among the environmental health hazards. Traffic is also a major source of CO_2 _and some other greenhouse gases.

In the Helsinki metropolitan area, there are about 4300 traffic accidents that kill 25 persons and injure 1300 persons every year [3]. The health and material costs in the metropolitan area are approximately 1 million € per day (227 million € per year in Helsinki alone [4]). In Finland, traffic accidents are the second most important cause of death in the age group 15–24 years [5]. It is therefore clear that road traffic is a major public health concern. However, it is also clear that one cannot envisage a modern society with no traffic. This does not mean that the problem can be ignored – research needs to be done on alternatives to current road use and ways need to be found to minimize the impact on public health.

There have been numerous efforts to reduce different kinds of impacts of road traffic, such as emissions (electric, hybrid, and hydrogen cars [6], natural gas buses [2], catalysts and particle traps [7], and driving style [8]); congestion (traffic control [9], street tolls, public transport subsidies); injuries (airbags, speed limits [10] and need to travel (urban planning [11]). Despite these efforts, the general view is that the environmental, health, and other detrimental effects of car traffic will continue to increase in the future. Although many modes of public transport are more efficient, they cannot compete with the flexibility of the private car. Thus public transport is not used as much as would be optimal for society. Novel systems that are both flexible and efficient should have special interest to municipal planners.

Several attempts have been made to develop transport based on trip aggregation. There exists car sharing clubs that rent cars to their members on a pay-as-you-drive basis even for periods as short as one hour. The renting of a car can often be done conveniently on the Internet or using a cell phone with the car being picked up from and returned to dedicated areas that are located around the city. These clubs can be economically viable without subsidies. They attract people that drive less than average, because the fixed costs are clearly lower than with car ownership. Club membership usually reduces car driving by 30–50% and increases the use of public transportation [12]. Such a club exists also in Helsinki.

In car pooling, people form groups that travel together instead of using their own individual cars. In many countries, car pooling is encouraged and there are dedicated lanes for cars with several passengers. Car pooling may be a practical solution for commuting in some cases, but it is not feasible for the majority of trips. There is no policy actively encouraging car pooling in Finland.

Municipalities have subsidised demand-responsive public transport in some places, such as the outlying areas of the Helsinki metropolitan area, or within the metropolitan area to organise trips for handicapped people. The former system was developed as a response to the disappearance of regular bus routes. Although important for some specific subgroups, these systems have handled only a small subset of all trips, and the low volume prevents efficient aggregation. Indeed, there have been complaints about poor service related to low volume: long waiting times and lengthy routes to the destination.

However, also positive results have been obtained from some pilot projects aggregating trips. In Finland, this has been the case with trips that are organised by the government or municipality due to societal reasons, such as some patient trips to and from hospitals, or lengthy school trips. Because society has paid the cost of the trip, it has also directly gained benefits from trip aggregation. The volume of these regional pilot studies has been in the order of 100 trips per day. [13]

In 2000 in the Helsinki metropolitan area, there were 2.9 million trips made every working day a region with one million inhabitants (the communities of Helsinki, Espoo, Vantaa, and Kauniainen). These trips were divided as follows, 1.3 million were by private car, 0.8 million by public transport, and 0.8 million on foot or by cycling [14]. In this work, we modelled the impact and potential of a hypothetical large-scale demand-responsive public transport system in the Helsinki metropolitan area, where a centralised system would collect the information on all trip demand online, aggregate the trips with the same origin and destination into public vehicles with eight or four passenger seats, and transmit the trip instructions directly to the passengers' mobile phones. We designate this system as *composite traffic*, as it represent a composite of the flexibility of the taxi and the efficient trip aggregation of the bus. We studied 1) how effectively trips could be aggregated; 2) what are the various costs and pressures of car and composite traffic; 3) what are the perceived costs for different passengers; 4) what incentives are needed to reach particular composite traffic volumes or areal coverage; and 5) how variation, uncertainty, and multiple decision-makers would affect the decision situation.

## Methods

The model was built using the Analytica 3.1™ program that utilises a graphical interface for creating probabilistic (Monte Carlo) models . It consists of two parts: first, a deterministic trip aggregation model that produces the output tables used in the decision analysis. The calculation of the results takes several days and therefore is calculated separately. In the second part, the trip aggregation results are combined with unit cost functions, emission factors and other uncertain and/or varying variables using a probabilistic simulation.

**Figure 1:**
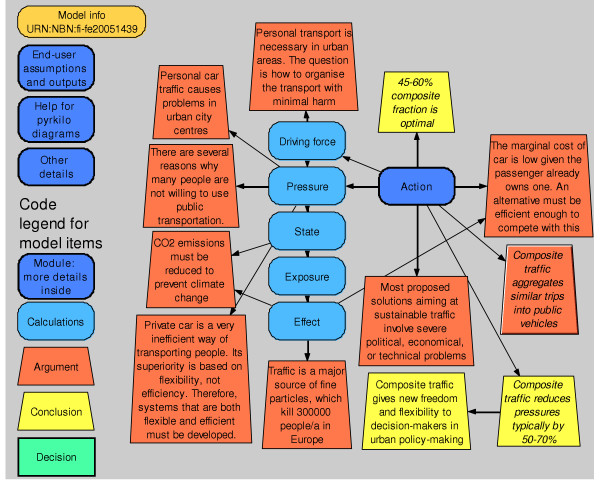
**Overview of the model *Composite traffic v. 1.0.1***. The model calculates health effects and other costs in the Helsinki metropolitan area. The overview of the urban traffic problem utilizes the DPSEEA approach (driving force, pressure, state, exposure, effect, action) [22]. The most important colour and shape symbols are explained in the lower left corner.

One of our major aims was to create the model in such a way that a non-specialist could follow the logic, reasoning, and conclusions without going into the modelling details (Figures 1, 7, and 8). In addition, he/she should even be able to test the model by using some personal assumptions. To facilitate this, we used a system diagram method denoted *pyrkilo *(see [2,15] and supplemental material at ). It has been developed in KTL as component of the science-policy interface, i.e. promoting the flow of information and understanding between science and policy, within the field of environmental health.

The principle is to describe an environmental health risk situation in a formal manner utilising system diagrams with causal connections of actions, outcomes, and interconnected variables (for causal diagrams, see [16]). As an example, a pyrkilo diagram may contain items along a causal pathway from abatement strategies for emissions to their dispersion to exposure to health effects (blue ovals, see Figure 1). However, the diagrams also describe parameters other than causal connections such as values, preferences and arguments, and finally conclusions from the examination (orange and yellow blocks). Arrows depict causal or non-causal connections between items. One novel feature of the pyrkilo diagrams is that they include and deal with three different kinds of variables in a single examination. These are physical variables related to a health hazard, political variables related to valuation of the outcome and other issues, and variables related to the assessment process itself, such as scope and conclusions.

The pyrkilo diagrams used in this study are based on an assessment of the literature by the authors. Stakeholders were not involved in this first study. However, the problems of and solutions to urban transportation have practical implications in everyday life. Stakeholder studies would therefore be useful in the future to describe and understand important issues and value judgements related to composite traffic and other proposed solutions.

The whole metropolitan area was divided into 129 areas. The 129 areas are standard areas used in urban planning and they contain on average 7300 inhabitants (standard deviation 5000 inhabitants). We used a road matrix containing 234 major links between the 129 areas; there was only one link connecting any two neighbouring areas, and there was exactly one, pre-specified route between any two areas.

### Trip data and trip aggregation

We took modelled personal car trips (public transportation, cycling, and walking were excluded from this exercise) for one working day (year 2000) into our model. Trip rates were estimated for each origin-destination pair (129*129 pairs) and for each time point (12 min intervals, resulting in 120 time points) based on summary data of trips in the Helsinki metropolitan area [14]. The summary data was disaggregated into smaller areas using numbers of population and jobs in each area [17]. The trips were disaggregated over 24 hours based on time activity data in the traffic (based on diaries) [18].

All scenarios had the same street structure and number of trips with a particular origin, destination, and time. The trips were divided into car trips and composite trips differently in each of the 91 scenarios based on 1) the percentage of the trips that are handled by composite traffic *(composite fraction) *ranging from 0 to 100% (13 alternatives; default: 50%), and 2) the area where composite traffic would be provided (i.e. the area within which a composite vehicle is guaranteed if desired) ranging from downtown Helsinki (81810 inhabitants) to the whole metropolitan area (944200 inhabitants) (7 alternatives; default: the whole area). The number of cars driving and kilometres driven by personal cars were calculated assuming 1.5 passengers per car.

The composite traffic trips were allocated into public vehicles with either eight or four passenger seats. The trips were aggregated if they had the same origin and destination areas (129 areas) and timing (12 min intervals). If there were less than four passengers in an aggregate of trips, the trips were divided into two parts, and the passengers had to transfer into another vehicle at the most busy point along the route. This made it possible to effectively aggregate trips with the same origin but different destination and vice versa. If there were still not enough passengers to fill a vehicle, a non-full vehicle was used; everyone was guaranteed of receiving a ride.

We assumed that the composite traffic would be a door-to-door service, or within walking distance comparable to that from a parking lot. The vehicles drove directly from the origin area to the destination area without stopping.

The actual trips, modes of transportation, and delays during trips and vehicle transfers were calculated, as well as the kilometres travelled by each type of vehicle and the number of vehicles needed.

### Decision analysis

In the decision-analytic part of the model, several of the outcomes modelled (i.e., pressures) were monetised and combined using probabilistic simulation. We assessed separately the uncertainty of an input value and the variation of the value between individuals in the population. Costs were separately calculated for the passenger and society. Some costs affect these stakeholders differently, such as fine particle and carbon dioxide emissions: these were calculated as societal costs only, not as costs to a passenger.

Estimating the costs and benefits of and human behaviour in a hypothetical traffic system is a difficult task. We used current values (including estimates on their uncertainties) whenever available, and wide confidence intervals otherwise. We made a special effort in trying to quantitatively estimate the individual variation within the population. Variation between individuals was separately estimated for three variables: how passengers evaluate the capital costs of owning a car; how passengers are willing to pay for either the right to drive themselves or the right not to drive; and how many passengers are travelling together.

The detailed descriptions of cost elements can be found in Table 2.

**Table 2:** The input variables used in calculations of the pressures. In most cases, there is no data available on uncertainty, and it is based on author judgement (AJ).

**Title, Unit [Reference]**	**Description**	**Definition**
Accidents, cases/a [3,24]	The number of injuries and deaths in traffic accidents in the Helsinki metropolitan area. Poisson distribution is used to describe the uncertainty.	Injuries: Poisson(1129) Deaths: Poisson(26)
Accident costs, €/d [24,25]	The societal costs of traffic accidents were 227 million euro in Helsinki in 2004. The numbers are scaled up from Helsinki to the metropolitan area based on the numbers of people injured in accidents. The uncertainty is based on the standard deviation of the variable Accidents (deaths), which is ca. 20% of the mean. We assume that half of the accidents are attributable to private car traffic, while the other half is attributable to other traffic modes (walking, cycling, public transportation). In addition, the accident risk is proportional to the change in traffic volume, but there is uncertainty about the slope. The expected value is that when traffic volume decreases by 10%, accident risk decreases by 5%; but it could vary between 0% and 10% (the latter being the default assumption in the guidelines for road construction planning).	var a:= 227 M*((1129)/724)/365; a:= normal(a,a/5) var b:= vehicle_km; b:= (1-b/b[comp_fr=0])*triangular(0,0.5,1); b:= (1-b)*a*0.5*vehicle_km
Vehicle price, €/vehicle [26]	Price of a new vehicle. Note that the interpretation is slightly different with different vehicles. The car price is the price that a random new car would cost, and it has therefore large uncertainty. The price of a composite vehicle is the average price of a taxi-style car in Finland, and the confidence intervals are narrower because there is no individual uncertainty. This is because the price of an individual car affects the costs of individual car trips, while the cost of a composite trip is dependent on the total cost of the fleet to the service provider. The same typical vehicles are used as in the Emission factor.	8-seat vehicle: 39520*Triangular(0.75,1,1.25) 4-seat vehicle: 22600*Triangular(0.75,1,1.25) personal car: lognormal(19100,1.5) [median, geometric standard deviation for lognormal distribution]
Vehicle lifetime, a Author judgement (AJ)	Expected operation time of a new vehicle.	8-seat vehicle: 7*Triangular(0.75,1,1.25) 4-seat vehicle: 5*Triangular(0.75,1,1.25) personal car: 9*Triangular(0.7,1,1.3)
Cap variab, fraction (AJ)	The value a car-owner gives to capital costs of the car as a fraction of the true costs. Each row represents one possibility for the distribution of individual valuations in the population. Probability distributions are used to represent this variation within the population.	Three possible distributions of variation within the population: A: Uniform(0,1) B: Triangular(0,0,1) C: Bernoulli(0.2))
Cap uncert, – (AJ)	The uncertainty between several valuation distributions about Cap variab on the population level.	A: 1/3 B: 1/3 C: 1/3
Trips per car, trips/d/car (AJ)	Average number of trips per car per day, i.e. the cumulative number of passengers that use the car during the day. This value is used to calculate the vehicle capital costs.	uniform(4,10)
Parking space, €/d/parking space [25]	Cost of a parking space to society due to the loss of the land, and maintenance costs. The average price of development land in Helsinki is around 300 €/m^2^, and one parking space requires ca. 20 m^2^. The standard values in road planning are 30 years for scope and 5%/a for discount. Opportunity cost for land is calculated based on these values; in addition, it is assumed that 50% of composite traffic parking places can be located in areas where the parking cost is negligible.	9.1*lognormal(1,1.3)
Parking price, €/trip [27]	The cost of 30 min parking in zones 1, 2, 3 in Helsinki. It is assumed that each car trip involves 30 min of parking during daytime, while during evening and night, the parking is free. Also daytime parking at home is included in these estimates, although it is difficult to price. In any case, it is common to pay at least 5–10 euro per month for a parking place (or more for a garage), which is 15–30 cents per day. Due to the uncertainties, the confidence intervals are large.	Downtown: 2.4*0.5*Triangular(0,1,2) Other centre: 1.2*0.5*Triangular(0,1,2) Suburb: 0.6*0.5*Triangular(0,1,2)
Emission factor, g/km [26,28]	Fine particle and carbon dioxide unit emissions for average vehicles. Fine particle emissions are taken from the Lipasto model using average (mixed gasoline and diesel) values for personal car and diesel EURO3 (applied since 2000) values for composite vehicles. For CO_2_, typical emissions of a new car were used based on the Finnish Vehicle Administration AKE. The following vehicles are used as typical examples of the class: 8-seat vehicle: Toyota Hiace 2.5 D4D 100 4 door long DX bus (diesel) 4-seat vehicle: Toyota Corolla 2.0 90 D4D Linea Terra 5 door Hatchback (diesel) Car: Toyota Corolla 1.6 VVT-i Linea Terra 5 door Hatchback (gasoline)	var a:= triangular(0.3,1,1.7) var b:= triangular(0.9,1,1.1) PM emission: 8-seat vehicle: 0.1*a 4-seat vehice: 0.1*a personal car: 0.047*triangular(0,1,2) CO2 emission: 8-seat vehicle: 232*b 4-seat vehicle: 153*b personal car: 168*triangular(0.3,1,1.7)
PM unit lethality, deaths/kg [2]	Primary fine particle emissions of 24290 kg/a caused 12.5 deaths in a risk assessment study in Helsinki (Tainio et al 2005). We use the distribution of deaths per emission derived from that study.	fractiles([-722.3, 5.640, 42.28, 59.87, 80.13, 115.0, 203.7, 293.9, 359.8, 413.2, 464.0, 513.9, 566.2, 623.3, 685.4, 757.7, 844.1, 951.9, 1093, 1314, 2805])/1 M
Emission unit cost, €/kg [1,2,25]	The value of a statistical life is 0.98 – 2 M€ (Watkiss et al. 2005). CO_2 _emission trade started in the EU this year, and the market price is used. According to newspapers Helsingin Sanomat (May 7, 2005) and Taloussanomat (July 11, 2005), the price has varied between 10 and 30 €/ton. The standard road planning value for CO2 emission is 32 €/ton.	PM emission cost: PM_unit_lethality*uniform(0.98 M,2 M) CO2 emission cost: uniform(5,40)/1000
Driver salary, €/h [29] Statistics Finland 2005	Monthly salary and social security costs (35%), and scaled to one hour assuming 160 hours of work per month. The salary is based on that of bus drivers in municipality-owned bus companies.	var a:= 2313/160*1.35; normal(a,a*0.18)
Fuel consumption, l/km [26]	Fuel consumption of a vehicle. It is assumed that composite vehicles use diesel fuel and cars use gasoline. The values are based on standardised European consumption values of a new car. The same typical vehicles are used as in the Emission factor.	8-seat vehicle: (8.7/100)*Triangular(0.75,1,1.25) 4-seat vehicle: (5.7/100)*Triangular(0.75,1,1.25) personal car: (8/100)*Triangular(0.5,1,1.5)
Fuel price, €/l (AJ)	Diesel fuel price for composite vehicles; gasoline price for cars. The values are based on a general follow-up of retail prices in Finland in fall 2004 – summer 2005.	diesel: 0.95*triangular(0.8,1,1.2) gasoline: 1.22*triangular(0.8,1,1.2)
Car maintenance, €/km [30]	Maintenance costs (service, tyres, oil etc.). This is based on Autoliitto's report 'Costs of car 2004'. Insurance and use tax are excluded. Similar to capital costs, there may be other reasons to own the car, and then these would be sunken costs. Original values (assuming an old car with the original price 20000 e, 20000 km/a of driving) (€/a): Maintenance 844 Tyres 320. Thus, 1164/20000 = 0.0582 €/km	Triangular(0.03, 0.058, 0.086)
Ticket, €/trip (AJ)	The income that the service provider wants to receive from composite traffic users in addition to the price of the direct costs (vehicle, fuel, driver, and parking costs).	uniform(0.2,0.4)
Rush delay h/trip, fraction (AJ)	Delay that is caused by increased link intensity. The node contains two values. Delay is the average time of delay due to traffic jams during daytime. Reduction is the relative reduction to 'Link intensity' (average vehicle flow on the 30 most busy roads at 8.00–9.00 AM) that is needed to reduce the delay to 0 min.	Delay: Uniform(0,10)/60 Reduction: 0.3
Time unit cost, €/h [25]	The cost of time spent waiting for a composite vehicle or in traffic jam. This is based on the standard road planning values.	Triangular(0,5.9,11.8)
Drive variab, fraction (AJ)	Willingness to drive. This is expressed as fraction of composite driver's salary. Each row represents one possibility for the distribution of individual valuations in the population. Probability distributions are used to represent this variation within the population.	Three possible distributions of variation within the population: A: Uniform(-0.3,0) B: Triangular(-0.1,0,0.3) C: Uniform(-0.2,0.2)
Drive uncert, – (AJ)	The uncertainty between several valuation distributions about Drive_variab on the population level.	A: 1/3 B: 1/3 C: 1/3
Car occupancy, fraction [31]	Proportion of cars with different numbers of passengers. The original data is from streets entering downtown Helsinki during one weekday (from 6.00 to 21.00) in May.	Passengers (incl driver): 1: 0.72 2: 0.233 3: 0.033 4: 0.01 5: 0.004

## Results

**Figure 2:**
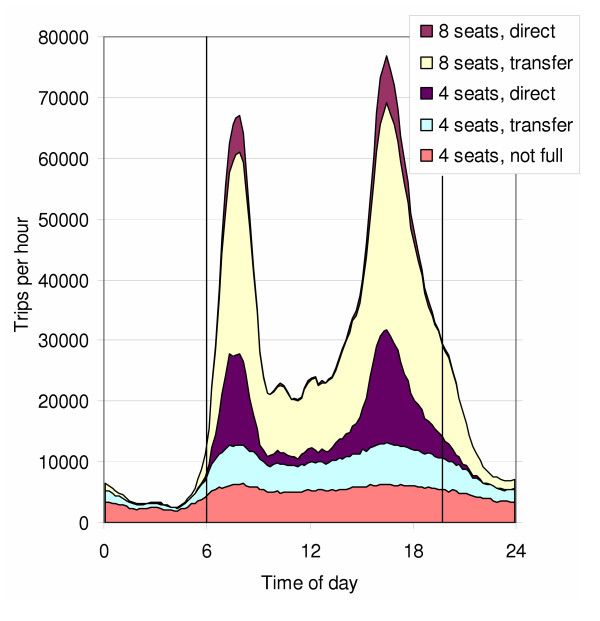
**Composite traffic trips by vehicle type as a function of time**. The fraction of composite trips *(composite fraction) *is 50% of the current 1.3 million personal car trips per day. Note that a trip with a transfer is calculated as two half-trips and may appear in two different vehicle types.

The trips and vehicle types used are shown in Figure 2. The need for less efficient vehicles (4-passenger-seat vehicles not fully occupied) remained almost constant in spite of the large variation in the trip volume occurring during the day. When the volume increased, more efficient vehicles were used, and therefore the number of vehicles increased in a sublinear fashion. In addition, the fraction of trips without transfer increased. The improved performance during rush hours is a major benefit compared with personal cars, for which the opposite is true.

The several outcomes studied clearly indicated that composite traffic would reduce most pressures attributable to private car (Table 1). For example, large reductions were seen in traffic volumes during rush hours (this is of benefit to car drivers as well) and CO_2 _emissions. (Note that the absolute numbers of vehicle flow are likely biased upwards because most smaller roads are excluded from the model.)

**Table 1:** The pressures and costs from traffic (composite+car) in the Helsinki metropolitan area.

Pressure	Private cars only	25% composite traffic	50% composite traffic	75% composite traffic	100% composite traffic
Fraction of composite trips without transfer (%)	-	8.7	19.5	28.1	35.0
Vehicles needed (number)	68000	60700	49300	37000	19700
Parking spaces needed (number)	91900	81900	66800	49600	25200
Average vehicle flow on the 30 most busy roads (vehicles/h at 8.00–9.00 AM)	5150	4350	3440	2380	1220
Injuries due to accidents (cases per year)	565 (537–593)	532 (498–566)	483 (425–543)	429 (336–524)	367 (233–504)
Deaths due to accidents (cases per year)	13.0 (9.0–17.5)	12.2 (8.4–16.3)	11.1 (7.52–15.2)	9.89 (6.35–14.0)	8.46 (4.66–12.9)
Deaths due to fine particles (cases per year)	95.4 (0.3–292)	96.7 (0.6–284)	87.6 (0.6–253)	75.8 (0.5–215)	61.0 (0.4–179)
Fine particle (<2.5 μm of diameter) emissions (kg per day)	500 (158–842)	507 (239–774)	459 (258–659)	397 (242–551)	320 (167–473)
Carbon dioxide emissions (ton per day)	1790 (1660–1910)	1580 (1480–1670)	1280 (1210–1350)	953 (907–999)	574 (535–613)
Driver salaries (thousand € per day)	0	599 (422–776)	947 (667–1230)	1260 (888–1630)	1560 (1100–2020)
Vehicle costs (capital+operational) (thousand € per day)	2750 (1930–3930)	2340 (1710–3230)	1820 (1380–2430)	1270 (1030–1590)	667 (582–753)
Time cost due to delay (thousand € per day)	365 (20.7–994)	308 (77.8–664)	233 (73.8–393)	328 (104–553)	424 (134–713)
Average car trip cost to passenger (€ per trip)	2.88 (1.74–4.19)	2.76 (1.65–4.02)	2.66 (1.57–3.94)	2.76 (1.62–4.09)	-
Average composite trip cost to passenger (€ per trip)	-	3.70 (3.00–4.42)	2.91 (2.38–3.43)	2.68 (2.19–3.15)	2.54 (2.08–2.99)

Mean (90% confidence interval when applicable). The number of vehicles and parking places is theoretical and involves the modelled trips only; a car owner may need the car for trips outside Helsinki even if he/she uses composite traffic. The true number of cars in the area was 346 400 in 2001 [14]. Decreased congestion reduces the costs of private cars. This feedback phenomenon is partly taken into account in time costs. The current ticket prices for buses, subway, and trams are 1.70 € per trip in Helsinki and 2.90 € per trip between communities in the Helsinki metropolitan area. Note that the car trip and composite trip costs include time costs due to delay (rush, transfers). If a passenger requires a trip without a transfer, the additional price to him/her will be 3 – 6 € per trip during daytime. This cost is due to reduced efficiency in trip aggregation.

**Figure 3:**
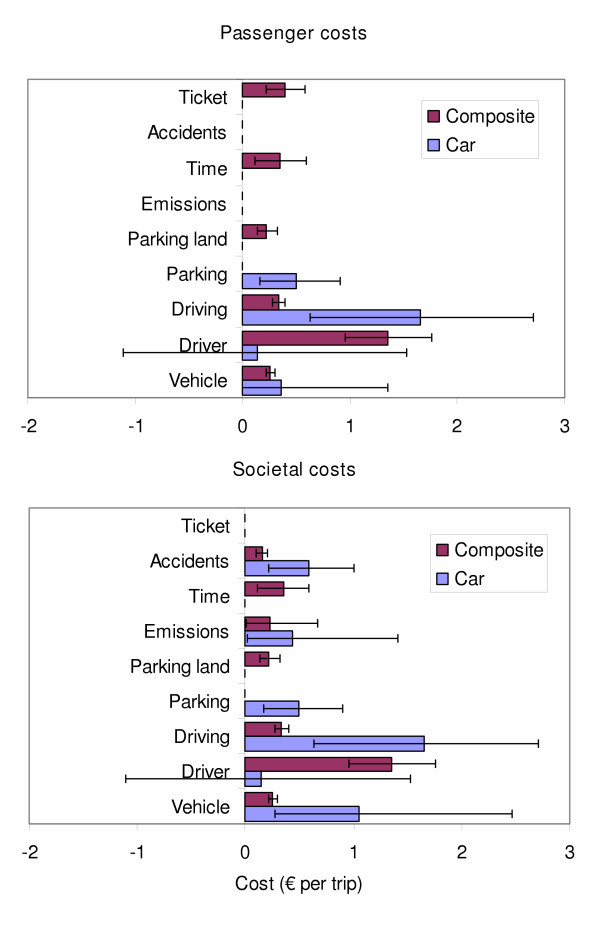
**Costs of daytime trips separated by source (mean and 90% confidence intervals)**. Emission costs are not calculated for the passenger; ticket costs are not calculated for society. Driver costs with car may be negative, because some people prefer driving themselves. There are no time costs for cars in the figure, because it was estimated that there are no traffic jams in the default scenario (50% composite traffic).

Fine particle emissions did not decrease as extensively due to the putative shift from the current gasoline-dominated car fleet to diesel composite vehicles. Improved technology may change this result in the next few years [6]. For example mandatory particle traps in all new diesel vehicles would dramatically reduce the fine particle emissions from composite traffic. However, at the level of 50% composite traffic, there would be about ten fewer deaths due to accident and PM emission reductions, which is not negligible. The salary of the driver was a new, expensive cost item. It is the main reason why the composite traffic is not obviously superior to the private car (Figure 3).

Of all trips in Figure 2, 20% were direct; but 80% did involve one transfer at one of the specific transfer points but with only a few minutes of extra waiting. The willingness to use public transport generally decreases if there is a need to change once or especially more than once. With composite traffic, this discomfort is likely to be less of a problem because even with one transfer, the shortest route is used and the impact on trip duration is minor.

**Figure 4:**
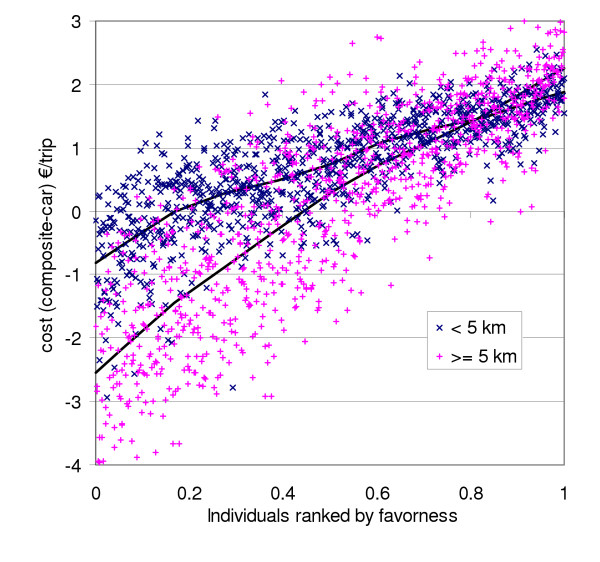
**Individual variation and uncertainty in the cost of a composite trip**. The cost of a composite trip is compared with a private car trip for an individual passenger. The estimates are for daytime trips with 50% composite fraction scenario. The trips are divided into two groups based on length (blue cross: <5 km; red plus: >= 5 km). The variation between individuals is shown on the X axis, with people most in favour of composite traffic on the left. The expected values across individuals are shown as lines, and the dots represent the uncertainty of the value.

One relevant stakeholder that should be considered is the random passenger (i.e., not average) who could take a car but would consider composite traffic as an alternative. The overview of this modelled examination is shown in Figure 4. Almost half of the modelled daytime passengers were estimated to find composite traffic more attractive than the private car even without any subsidies. The percentage was 45% for trips longer than 5 km (these trips consist of 75% of all trips), and 20% for shorter trips. The single most important variable affecting the attractiveness was the number of persons travelling together, as this obviously reduced the cost per person with the private car.

**Figure 5:**
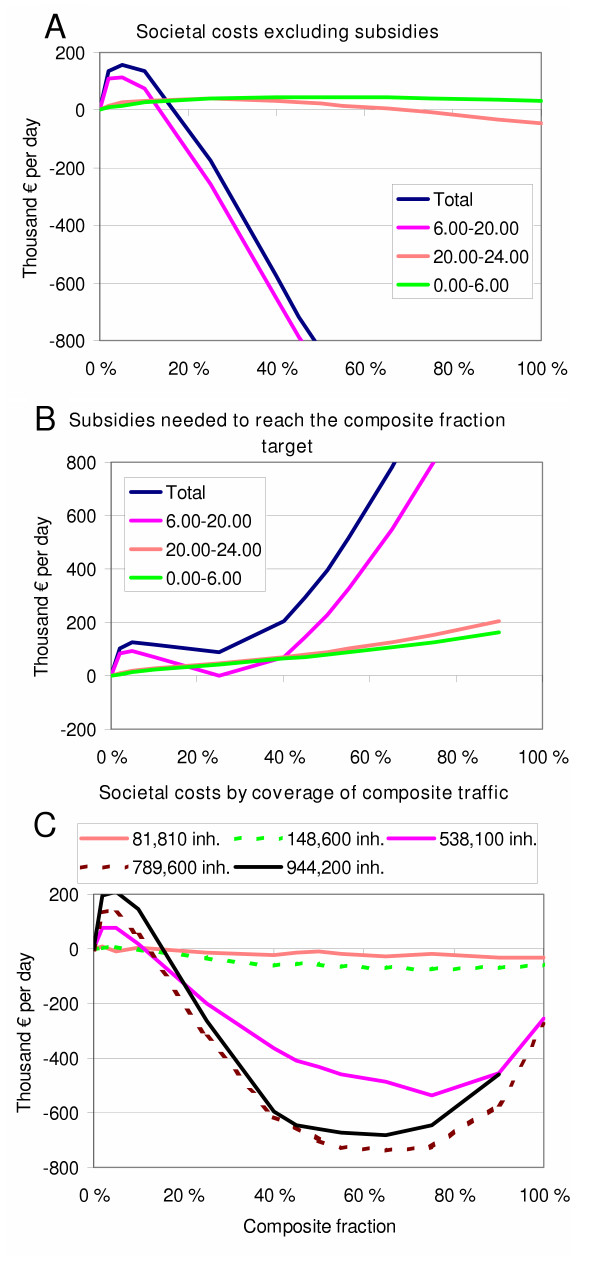
**Marginal societal costs of traffic (composite+car) as a function of composite fraction**. **A**: Societal costs (excluding subsidies for composite traffic) during different periods of day. **B**: Subsidies to composite ticket prices needed to reach the composite fraction target (i.e., to make that fraction of current private car passengers to favour composite traffic according to the model) during different periods of the day. For comparison, the current subsidies to public transportation in the Helsinki metropolitan area are on the range of 380 000 € per day [23]. **C**: Societal costs (including subsidies) during daytime with extending areal coverage of composite traffic (starting from the most densely populated areas). The legend shows the number of inhabitants living in the covered area. The pink curve (538100 inhabitants) is the city of Helsinki).

Another stakeholder is society: whether it should subsidise composite traffic to achieve a certain level of modal shift from private cars to composite traffic. Society is burdened also with different costs compared to those incurred by the individual passenger: health effects of fine particles, greenhouse gas emissions, and opportunity costs for land currently used as parking spots. Figure 5A and 5B show that composite traffic is clearly beneficial when it replaces 30% or more of the private car traffic. Although the societal cost (excluding subsidies) decrease until all car traffic is replaced (Figure 5A), the subsidies needed to attract more passengers to composite traffic increase progressively (Figure 5B). It seemed that at 70–80%, the subsidies start to have negative impacts on the societal benefits.

Another important practical question is whether it is possible to start up a system like this without prohibitive capital expenditure. As we have pointed out, composite traffic becomes more profitable when there is a high volume of trips. Thus, it seems that a good strategy would be to start the operation during heavy commuting hours in densely populated areas, and then to rapidly expand the service beyond the low-volume "inefficiency bump" seen in Figure 5A. A volume that would be already profitable to the service provider and still produce societal benefit is 8000 trips per day (25% composite fraction during daytime in downtown Helsinki, an area with 82000 inhabitants) (Figure 5C). This would require a fleet of about 250 vehicles, which is 10% of the number of taxis currently in the Helsinki metropolitan area [19]. If this critical volume is reached, the system becomes more and more profitable as it expands, at least until it covers around 50% of all daytime car trips in most of the area (420000 trips per day).

We used several origin-destination trip matrices to test the sensitivity of the aggregation. One extreme case tested was a matrix where the trip rate from all areas to all other areas was the same number at each time point. The total volume at each time point varied in the same way as in the default matrix. This is a very flat trip matrix with no spatial trip aggregates, which is an unfavourable situation for the composite traffic. However, the major conclusions remained the same with all matrices tested. The largest differences between the matrices were that the fraction of trips without transfer decreased with flat matrices, and the number of vehicles needed varied, usually being higher than with the default matrix.

The model did not directly assess the additional driving needed to pick up or drop off passengers. Therefore we performed a sensitivity analysis where the composite trips were on average 1.8 km longer than the private car trips (this was a rough upper-bound estimate based on the sizes of origin and destination areas). On average, this increased the cost by approximately 20 cents per trip. Although this is not negligible, it does not alter the overall conclusions (compare for example to Figure 4). Further studies are warranted on this issue.

**Figure 6:**
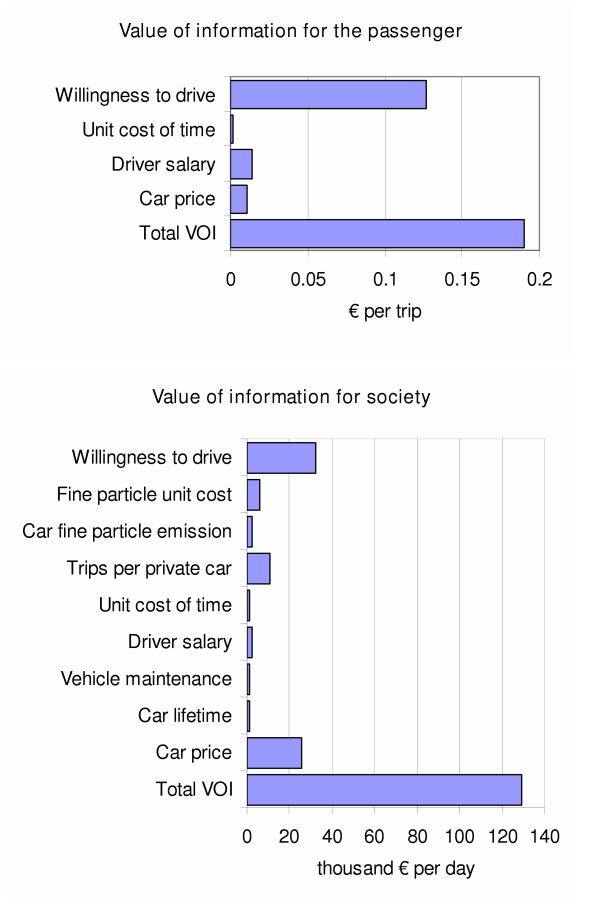
**Value of information of the daytime trips for society and the passenger**. *Total VOI *is the expected value of perfect information for all uncertainty; other rows are expected values of partial perfect information for each uncertain variable. Variables with zero value (i.e. variables that could not change the decision) are omitted. A part of the value of car price is actually due to variation that is not explicitly separated from the uncertainty.

The separate treatment of uncertainty and variation made it possible to evaluate their importance in the model. The value of information (i.e. the price that is worth paying to reduce a particular uncertainty) was calculated for these decisions [20]. For the passenger, there is little value of information, as there is mainly individual variation and only marginal uncertainty (Figure 6A). For the societal question of whether to subsidise composite traffic at 50% composite fraction or not at all, the total value of resolving all uncertainty is only about 30 000 € per day, and the value for every single variable was zero. This means that the conclusion is robust and even if the certainty about any single variable was found out to be the most unfavourable to the composite traffic, the optimal decision would still remain unchanged. However, when the question was about the optimal level of subsidies, more uncertainty was involved with the decision (Figure 6B). The single most important variable was about the customers' willingness to drive him/herself rather than being a passenger in a public vehicle. This suggests that comparative studies on passenger attitudes are warranted.

**Figure 7:**
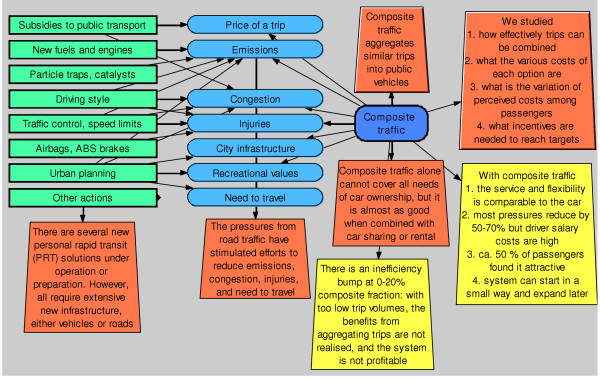
The Action module.

**Figure 8:**
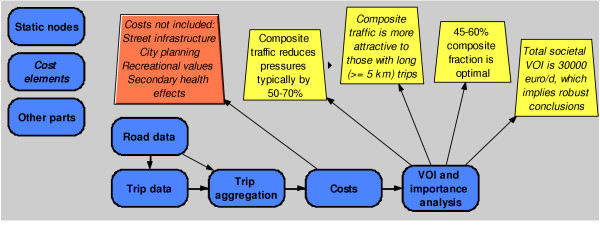
The Composite traffic module.

## Discussion

Compared with buses and the subway, the composite traffic is quick because it uses the shortest route and does not stop between the origin and the destination. Nonetheless, it is likely to be slightly slower than the private car, because a few minutes are lost during transfer; in addition, the vehicle picks and drops 4–8 passengers from and to the ends in a limited area. On the other hand, it is often possible to transport the passenger directly to the destination without the need of searching for a parking place nearby.

We were careful not to unrealistically exaggerate the benefits of the composite traffic. On the contrary, we excluded several clear but not easily quantifiable benefits: Replacement of low-volume bus routes with composite traffic would improve service and reduce costs at the same time. Composite traffic would be an efficient feeder for high-volume public transport modes. Reduced road traffic volumes would save money currently used on road construction, maintenance, and infrastructure. Finally, we assumed that the trips are uncorrelated in time (given the total volume at each time point). However, trips are often directed to or from particular locations such as schools, offices, stadiums, and supermarkets at specific times, which improves trip aggregation.

There are several kinds of approaches in use to solve some of the problems attributable to car traffic. Some involve improving the car to reduce its pressures per vehicle-km (airbags or catalysts [7]); some aim at improving traffic flow [9]. There have also been attempts to replace the car by developing *personal rapid transit *– a transportation mode that utilises new techniques to offer flexible transportation services [21]. Although this is novel approach, these systems usually require extensive (and expensive) new vehicle or road infrastructure, and this lessens the possibilities of their implementation and success.

Composite traffic requires no new techniques, except a system for collecting, organising, and distributing the trip information. The interface to this database and optimising system could be based on text messages sent to mobile phones, in conjunction with an Internet site where passengers can create personalised profiles. The composite vehicles would probably need to have a real-time connection to a global positioning system and a centralized trip optimising system. This would enable a minute-to-minute planning of vehicle routes and destinations so that the number of vehicles driving empty or waiting could be minimised.

We did not estimate the capital or operational costs of implementing the trip optimising system, but it is reasonable to assume that it could be centrally organised by the community without dramatically changing the level of societal benefits. For comparison, the current electronic travel card system in Helsinki area handles a million trips per day with operational costs ca. 0.02 € per trip [3].

We did not aim to describe in detail, how the system would look like in practice. The eight and four-passenger-seat vehicles were used as examples of possible vehicles because they are currently in mass production. The fuel and propulsion systems could well be better than the current standards, because the relatively small fleet, small geographical operational area, and probably centralised ownership would allow for specialised solutions. The aim of this work was simply to study whether it would be possible to develop a flexible transport system that would utilise trip aggregation and that would be more effective than the current private car.

## Conclusion

In conclusion, we have shown that 1) with composite traffic, it is possible to aggregate trips in an urban area so that the level of service and flexibility are comparable to those achieved by private car; 2) many important pressures caused by car traffic could be reduced by 50–70% (Table 1), but at the cost of considerable driver salary costs; 3) almost 50% of day-time passengers would consider it more attractive than using their own car even without subsidies; 4) it is possible to start the service with a small volume and then expand it later, thus reducing the financial risks; and 5) the passenger and society have an important interplay as decision-makers, but the composite traffic shows robust benefits to both groups despite current variation and uncertainty.

However, the most important impact (and the most difficult to model) of composite traffic may come from the increased degree of freedom in urban policy-making and planning, and public health policy, when the pressures associated with increased private car use and traffic are relieved without impairing the ability of people to travel rapidly about the city.

## Competing interests

The author(s) declare that they have no competing interests.

## Authors' contributions

JTT conceived the study, performed modelling and drafted the manuscript. MT participated in the design of the study and helped to draft the manuscript. Both authors read and approved the final manuscript.

## Pre-publication history

The pre-publication history for this paper can be accessed here:



## Supplementary Material

Additional file 1 **Composite traffic model for calculating health and other pressures. **CompositeTraffic_1_0_1.ana (Analytica™ 3.1 file in XML format). Model identifier (unified resource name) is URN:NBN:fi-fe20051439. For a free browser, see . The model contains the full model including the code and the descriptions. It has the road and trip data algorithms as well as static (precalculated) results; the decision analysis can be performed in real-time using the default or user-defined assumptions. For more guidance and updates of the model, see . Click here for file
